# Thalidomide Inhibits Alternative Activation of Macrophages *In Vivo* and *In Vitro*: A Potential Mechanism of Anti-Asthmatic Effect of Thalidomide

**DOI:** 10.1371/journal.pone.0123094

**Published:** 2015-04-23

**Authors:** Hyun Seung Lee, Hyouk-Soo Kwon, Da-Eun Park, Yeon Duk Woo, Hye Young Kim, Hang-Rae Kim, Sang-Heon Cho, Kyung-Up Min, Hye-Ryun Kang, Yoon-Seok Chang

**Affiliations:** 1 Institute of Allergy and Clinical Immunology, Seoul National University Medical Research Center, Seoul, Korea; 2 Department of Internal medicine, Seoul National University College of Medicine, Seoul, Korea; 3 Department of Medical Science, Seoul National University College of Medicine, Seoul, Korea; 4 Department of Anatomy, Seoul National University College of Medicine, Seoul, Korea; 5 Department of Internal Medicine, Seoul National University Bundang Hospital, Seongnam, Gyeonggi-do, Korea; Mie University Graduate School of Medicine, JAPAN

## Abstract

**Background:**

Thalidomide is known to have anti-inflammatory and immunomodulatory actions. However, the effect and the anti-asthmatic mechanism of thalidomide in the pathogenesis of asthmatic airways are not fully understood.

**Objective:**

This study is designed to determine the effect and the potential mechanism of thalidomide in the pathogenesis of asthmatic airways using animal model of allergic asthma.

**Methods:**

Six-week-old female BALB/C mice were sensitized with alum plus ovalbumin (OVA) and were exposed to OVA via intranasal route for 3 days for challenge. Thalidomide 200 mg/kg was given via gavage twice a day from a day before the challenge and airway hyperresponsivenss (AHR), airway inflammatory cells, and cytokines in bronchoalveolar lavage fluids (BALF) were evaluated. The expression levels of pro-inflammatory cytokines and other mediators were evaluated using ELISA, real time (RT)-qPCR, and flow cytometry. CRL-2456, alveolar macrophage cell line, was used to test the direct effect of thalidomide on the activation of macrophages *in vitro*.

**Results:**

The mice with thalidomide treatment showed significantly reduced levels of allergen-induced BALF and lung inflammation, AHR, and the expression of a number of pro-inflammatory cytokines and mediators including Th2 related, IL-17 cytokines, and altered levels of allergen-specific IgG1/IgG2a. Of interesting note, thalidomide treatment significantly reduced expression levels of allergen- or Th2 cytokine-stimulated alternative activation of macrophages *in vivo* and *in vitro*.

**Conclusion:**

These studies highlight a potential use of thalidomide in the treatment of allergic diseases including asthma. This study further identified a novel inhibitory effect of thalidomide on alternative activation of macrophages as a potential mechanism of anti-asthmatic effect of thalidomide.

## Introduction

Allergic asthma is a chronic inflammatory disease of the airways. It is characterized by pulmonary eosinophilia, mucus hypersecretion, an increase in serum levels of allergen-specific IgE, and airway hyper-responsiveness (AHR) and Th2 cytokines, such as interleukin (IL)-4, IL-5 and IL-13 [1–5].

In asthma pathophysiology, the Th2 cytokine profile in asthmatic lung contributes to the appearance of alternatively activated or M2 macrophages. These macrophages also generate several proallergic factors, such as chemokines, chitinase-like molecules [6] and ‘found in inflammatory zone 1’(FIZZ1, also known as Relm-α) [7,8], which all contribute to the airway inflammatory and remodeling responses during asthma [9,10]. It has also been shown that asthmatics have higher percentages of macrophages expressing mannose receptor in bronchial biopsies than in healthy subjects [11]. In addition, severe asthmatics had higher numbers of IL-13-positive M2 macrophages in bronchoalveolar lavage fluid (BALF) as compared to healthy controls [12]. Markers of M2 macrophages are correlated with severity of allergic airway disease in humans and mice, suggesting that M2 macrophages contribute to the disease. Elimination of M2 macrophages by pharmacological interventions remarkably decreased the degree of airway inflammation [13].

Recently, it has been reported that thalidomide suppressed airway inflammation and airway hyperresponsiveness in a murine model of allergic asthma by us and others [14,15]. However, little is known about the effects of thalidomide on Th2 cytokines and the mechanism of thalidomide on the airway hyperresponsiveness and eosinophilic inflammation in allergic asthma. In this study, we demonstrated that thalidomide treatment before and after each challenge with ovalbumin (OVA) almost completely suppressed allergen-induced eosinophil-dominant airway inflammation, AHR, and Th2-deriven humoral responses in OVA-sensitized mice. It also suppressed the expression of a number of pro-inflammatory and Th2 cytokines including tumor necrosis factor (TNF)-α, IL-4, IL-5, IL-13, and IL-17. These results strongly suggest a potential use of thalidomide as a therapeutic agent for the treatment of asthma and other allergic diseases. In addition, we showed thalidomide had a potent suppressive effect on alternative activation of macrophages *in vivo* and *in vitro*, which revealed, for the first time, a novel regulatory role of thalidomide on alternative activation of macrophages as a potential underlying mechanism of anti-asthmatic effect of thalidomide.

## Materials and Methods

### Animals

Female BALB/C mice (18–20 g) were purchased from SLC Inc. (Hamamatsu, Kotoh-cho, Japan) and were maintained in specific pathogen-free conditions. Six weeks old female mice were used in all experiments. All experiments were performed with the approval of the Institutional Animal Care and Use Committee of the Institute (IACUC) of Laboratory Animal Resources at Seoul National University (SNU-09-1111-4).

### Sensitization and airway challenge

**Fig 1:**
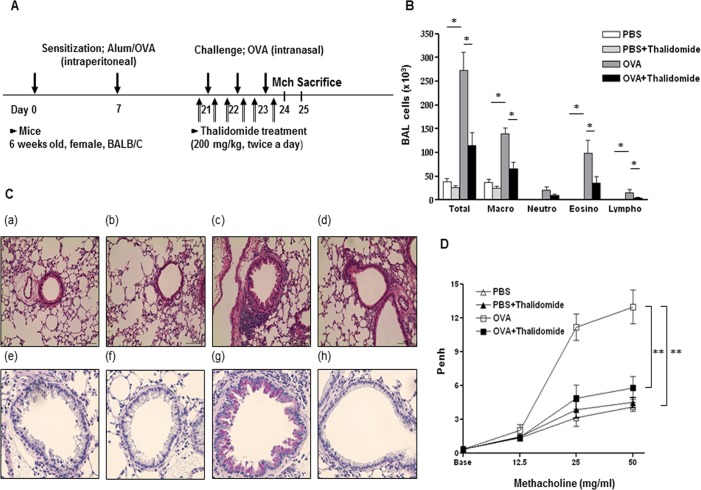
Effects of thalidomide on lung inflammation and AHR by allergen challenges. A, Experimental protocol of the study. B, The number of inflammatory cells including eosinophils in BALF. C, H&E stain (a-d), ×100) and PAS stain (e-h), ×200) of lung histology after allergen challenge (a,e: PBS; b,f: PBS+Thalidomide; c,g: OVA; d,h: OVA+Thalidomide). * indicates *p* < 0.05. (D) Methacholine hyperresponsiveness was measured 24 h after the last intranasal challenge, n = 5–8 for each group, the ** indicates *p* < 0.01. Mch; methacholine bronchial challenge.

BALB/C mice were sensitized by intraperitoneal injection with 75 μg of OVA (Grade V, Sigma Aldrich, St. Louis, MO, USA) and 2 mg alum (Pierce, Rockford, IL, USA) in 200 μL of phosphate-buffered saline (PBS) on day 0 and 7. Intranasal injection with 50 μg OVA in 20 μL of PBS was followed on day 21, 22, and 23 (Fig 1A).

### Thalidomide treatment

Thalidomide (Sigma Aldrich, St. Louis, MO, USA) was dissolved in 0.2 mL of 0.5% carboxymethylcellulose (CMC; Sigma Aldrich) and 0.25% Tween 80 (Sigma Aldrich). Thalidomide vortexed immediately before its use was administered orally by using a stainless needle 1 h before and 6 h after each of the OVA challenges in amount of 200 mg/kg (Fig 1A). The effective dose and frequency were selected based on the pharmacokinetic of thalidomide in mice [16]. The administration was repeated six times during challenge period. Control mice were orally administered vehicle.

### Measurement of methacholine hyper-responsiveness

One day after the last challenge with OVA, airway hyperresponsiveness was measured with a barometric plethysmographic chamber (OCP 3000, Allmedicus, Anyang, Korea) and the Penh (enhanced pause) value for 3 min was monitored.

### Analysis of bronchoalveolar lavage and serum

Twenty-four hours after the assessment of airway hyperresponsiveness, mice were sacrificed and bronchoalveolar lavage fluid and lung tissue were obtained. BAL fluid inflammatory cells were obtained as previously described [10]. At least 300 cells per slide were evaluated to obtain a differential leukocyte count. The supernatants were decanted and immediately frozen at -80°C. BAL cell slides were stained with Diff-Quik (Sysmex Co., Kobe, Japan). The level of IL-17 in BAL fluid was measured using commercially available ELISA Kit (Quantikine, R&D Systems Inc., Minneapolis, MN, USA) as manufacturer’s guideline. IL-4 and IL-5 in BAL fluid were measured using commercially available ELISA Kit (BioSource International, Camarillo, CA, USA). Serum OVA-specific IgG1, and IgG2a were measured by using ELISA.

### Histopathology

To evaluate and compare the severity and character of pathological changes in lung parenchyma, left lungs of mice were fixed in 10% neutral buffered formalin and embedded in paraffin, and 3-mm sections were stained with hematoxylin & eosin (H & E staining) and Periodic acid-Schiff (PAS) stain.

### Quantitative real-time RT-PCR

Total RNA was extracted from the whole lung. Total RNA (2 μg) from each sample was reverse transcribed into cDNA with a single-strand cDNA synthesis kit (Promega, Madison, WA, USA). Quantitative real-time PCR was performed on a 7500 Real-time PCR System (Applied Biosystems, Foster City, CA, USA). The mRNA expression of cytokines was determined by real-time PCR using SYBR Green Master Mix (Applied Biosystems). The expression of each gene within each sample was normalized against β-actin and expressed relative to the control sample using the formula 2-(ΔΔCt), in which ΔΔCt = (Ct mRNA—Ct β -actin). All used primer sequences were validated by Primer bank (Harvard, USA).

### Culture of cells

CRL-2456, alveolar macrophage cell line was purchased from the American Type Culture Collection (ATCC). Cells were grown in RPMI medium with 10% FBS and antibiotics (complete culture medium) in a humidified atmosphere at 37°C with 5% CO_2_, with refreshment of medium every 2–3 day. CRL-2456 were plated in 24-well plate (5 X 10^5^ per well), one day prior to exposure and then stimulated by LPS (1 mg/mL), recombinant IL-4 (20 ng/mL), recombinant IL-13 (20 ng/mL) and cultured for 18h in 24-well plate with or without the presence of thalidomide (100 μg/mL). Cells were analyzed using flow cytometry analysis. The mRNA expression was determined by Real-time RT-qPCR.

### Preparation of lung tissue, draining lymph nodes of lung, and spleen

Lung tissues from mice were minced and digested with 1.6 mg ml^−1^ of collagenase type 4 (Worthington, Lakewood, NJ) and 0.1% of DNase I (fraction IX; Sigma Aldrich) at 37°C for 1 h. Total cells were lysed for RBCs and stained for FACS analysis. Single cell suspensions of draining lymph nodes and spleen from mice were plated at a concentration of 1×10^6^ cells/mL onto a 96-well plate and re-stimulated with OVA protein (100 μg/ mL) for 72 hours for flow cytometry analysis.

### Flow cytometry

Single-cell suspensions were preincubated with FcγR-specific blocking mAb (2.4G2) and washed before staining. Cells were stained with the following antibodies Percp-cy5-conjugated anti-CD45 (eBioscience), APC-conjugated anti-F4/80 (eBioscience), PE-cy7-conjugated conjugated anti-CD11c (eBioscience), FITC–conjugated anti-CD206 (BioLegend), APC-conjugated anti-CD3 (eBioscience), FITC–conjugated anti-CD4 (eBioscience); For intracellular staining, cells were permeabilized (Cytofix/Cytoperm kit; BD Biosciences) and incubated with PE–conjugated anti-IL-4, PE–conjugated anti-IL-5, PE–conjugated anti-IFN-γ (eBioscience), rabbit anti-Ym-1 (Stem Cell Technologies) and PE–conjugated anti-rabbit IgG (eBioscience). Cells were analyzed on a LSRII (BD Biosciences) with FlowJo ver.10 software (TreeStar).

### Statistics

All data are expressed as mean ± standard error of the mean (SEM). Differences between groups were calculated for statistical significance using the unpaired Student’s *t*-test. *P* < 0.05 was considered as significant.

## Results

### Thalidomide inhibits eosinophilic inflammation, mucus secretion and airway hyperresponsiveness

WT mice were challenged with OVA intranasally and then sacrificed 48 h after the last challenge. While the number of total cells, macrophages, eosinophils, and lymphocytes in BALF was increased in OVA challenged mice, it was almost completely decreased by 200 mg/kg thalidomide treatment (p < 0.05) (Fig 1B). The recruitment of inflammatory cells into the lungs of mice was also investigated by histopathological studies. Control mice had no inflammatory cells in the lung (Fig 1C-a and b). OVA challenged mice showed infiltration of inflammatory cells around peribronchial and perivascular spaces (Fig 1C-c). The majority of the infiltrated inflammatory cells were macrophages and eosinophils. However, the infiltration of macrophages and eosinophils was significantly reduced in thalidomide-treated mice compared to OVA challenged mice (Fig 1C-d). In addition to inflammation, mucus secretion was reduced in thalidomide-treated mice compared to OVA challenged mice (Fig 1C-g and h).

We also examined the role of thalidomide in the development of AHR. AHR to methacholine was significantly increased in OVA challenged mice compared to control mice (p < 0.01). However, AHR was significantly reduced in thalidomide treated mice compared to OVA challenged mice (p < 0.05) (Fig 1D).

### Thalidomide alters the humoral immune response

**Fig 2:**
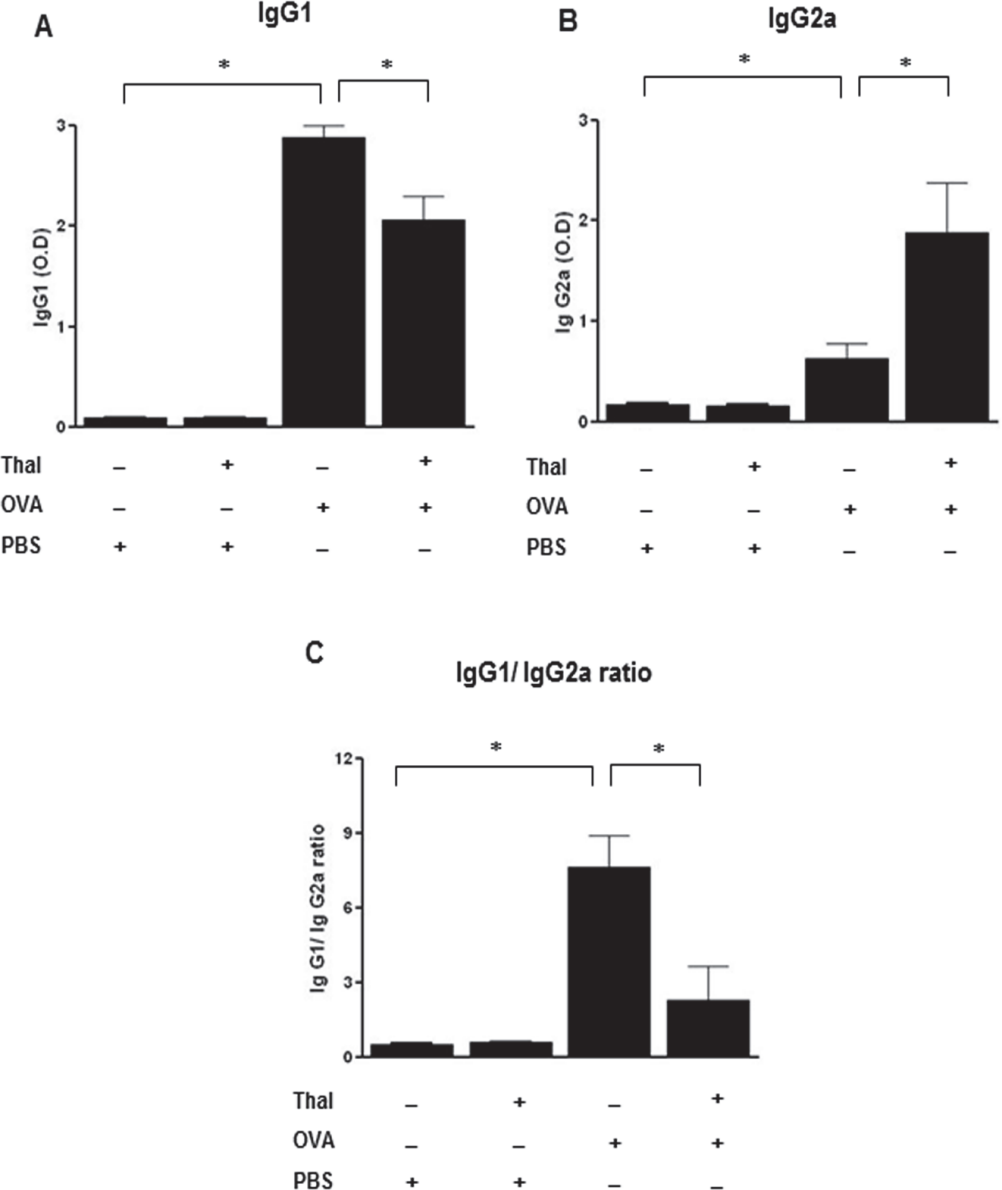
Effects of thalidomide on serum allergen-specific antibodies. A-C, The levels of serum OVA-specific IgG2a and IgG1 48h after the last challenge. Serum OVA-specific IgG1 (A), IgG2a levels (B) and IgG1/IgG2a ratio (C) after OVA challenge. n = 5–8 for each group, * indicates *p* < 0.05.

We examined the effect of thalidomide on OVA specific IgG1 and IgG2a antibody levels. The level of OVA specific IgG1 antibody was significantly increased in OVA challenged mice compared to control mice (p < 0.05). In contrast, The level of OVA specific IgG1 antibody was significantly reduced in thalidomide treated mice compared to OVA challenged mice (p < 0.05) (Fig 2A). Opposite effects were showed on OVA specific IgG2a antibody levels (p < 0.05) (Fig 2B). The ratio of OVA specific IgG1/IgG2a antibody was also significantly reduced in thalidomide treated mice compared to OVA challenged mice (p < 0.05) (Fig 2C).

### Thalidomide decreases IL-4 and IL-5 levels in BALF

**Fig 3:**
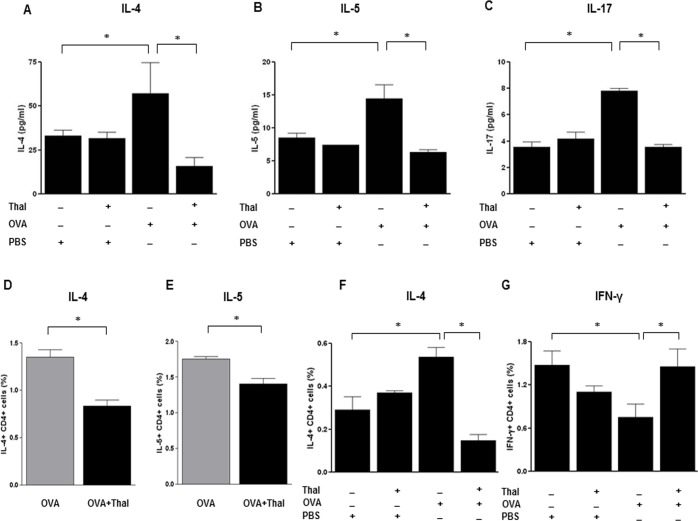
Effects of thalidomide on cytokine protein levels in BALF, Lung draining lymph nodes and spleen. A-C, The upper low shows the levels of IL-4(A), IL-5(B) and IL-17(C) 48h after the last challenge. The levels were detected with enzyme-linked immunosorbent assay. D-G, The lower low shows the frequencies of IL-4(D) and IL-5(E)-producing CD4^+^ T cells 72h after re-stimulated *in vitro* with OVA protein in draining lymph node cells. The frequencies of IL-4(F) and IFN-γ(G)-producing CD4^+^ T cells 72h after re-stimulated *in vitro* with OVA protein in spleen cells. The expressions were analyzed using Flow cytometry. n = 5–8 for each group, * indicates *p* < 0.05.

While the levels of IL-4, IL-5, and IL-17 from BALF were significantly increased in OVA challenged mice compared to control mice (p < 0.05), these levels were significantly reduced in thalidomide treated mice compared to OVA challenged mice (p < 0.05) (Fig 3A–3C). These data demonstrated that thalidomide inhibits the Th17 cytokine as well as the Th2 cytokines.

#### Thalidomide decreases IL-4 and IL-5-producing CD4^+^ T cells in lymph nodes and spleen

The frequencies of IL-4 and IL-5-producing CD4^+^ T cells in draining lymph nodes were significantly decreased in thalidomide treated mice compared to OVA challenged mice (p < 0.05) (Fig 3D and 3E). The frequency of IL-4-producing CD4^+^ T cells in spleen was also significantly decreased in thalidomide treated mice. On the contrary, the frequency of IFN-γ-producing CD4^+^ T cells in spleen was significantly increased in thalidomide treated mice compared to OVA challenged mice (p < 0.05) (Fig 3F and 3G).

#### Thalidomide decreases TNF-α, IL-13, Eotaxin-1, and MUC-5AC mRNA expressions in Lung

**Fig 4:**
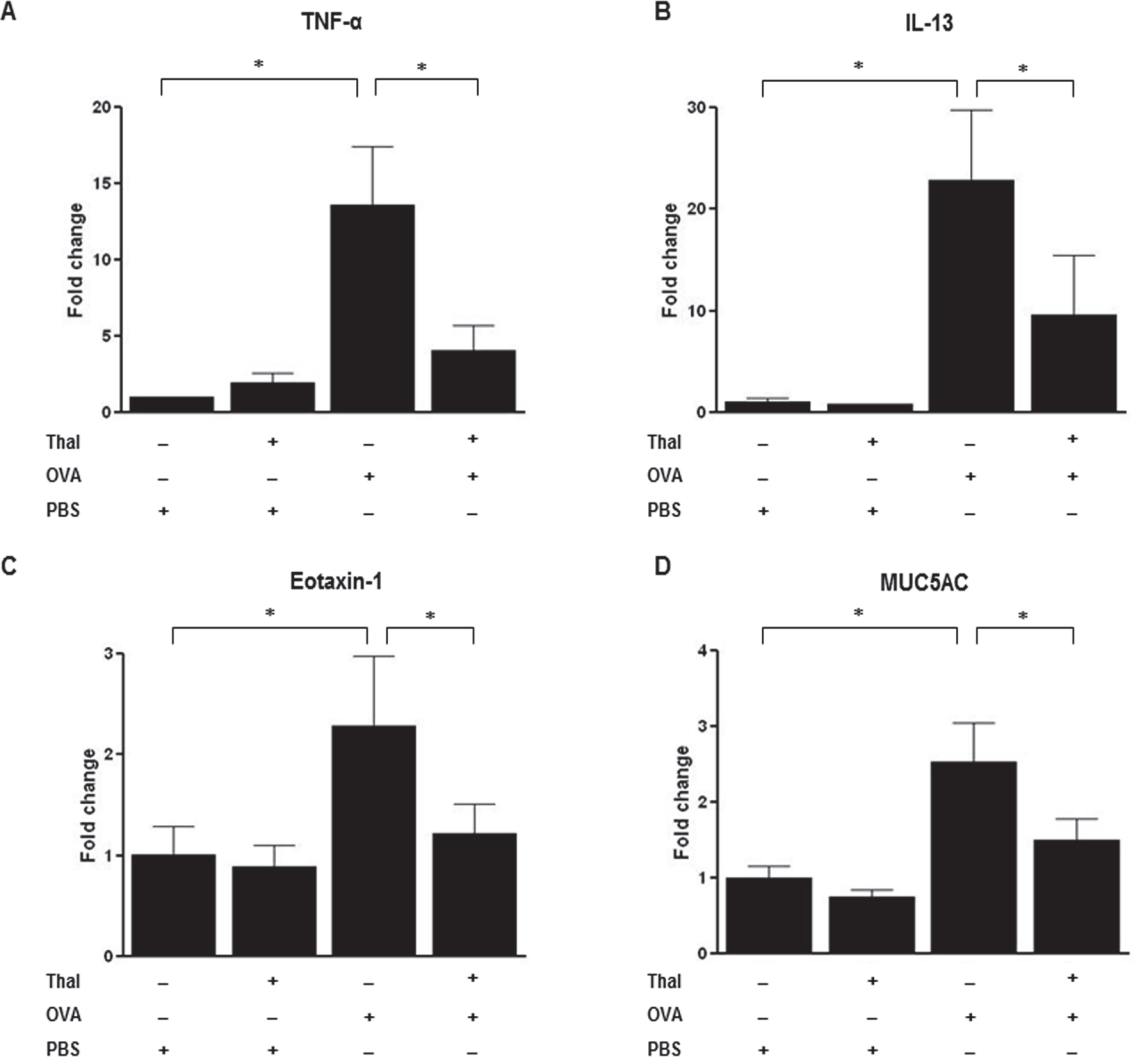
Effects of thalidomide on the mRNA expressions in mouse lung tissues. A-D, Real-time RT-PCR was performed to determine the changes in mRNA levels of TNF-α (A), IL-13(B), Eotaxin-1(C) and MUC5AC(D) from lung tissues. The levels of mRNA are represented as the ratio to β-actin. n = 5–8 for each group, * indicates *p* < 0.05.

Messenger RNA expressions were examined in lung homogenates. While analysis of mRNA expressions in OVA challenged mice revealed increased levels of TNF-α, IL-13, eotaxin-1, and MUC-5AC compared to control mice (p < 0.05), these mRNA expression levels in lungs were significantly decreased by thalidomide treatment in OVA challenged mice (p < 0.05) (Fig 4A–4D).

#### Thalidomide inhibits *in vivo* and *in vitro* expressions of M2 macrophage

**Fig 5:**
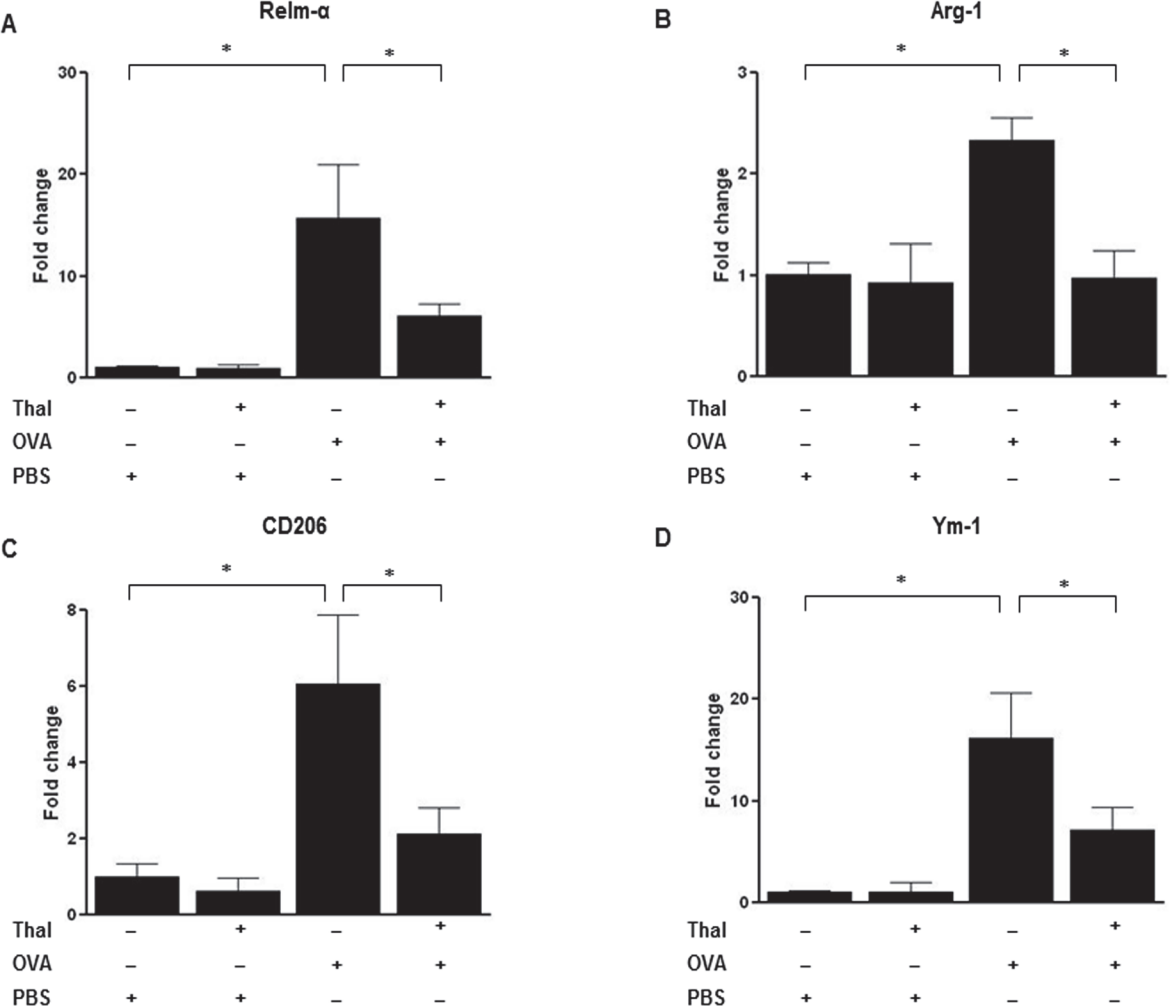
Effects of thalidomide on the mRNA expressions of M2 macrophage markers in mouse lung tissues. A-D, Real-time RT-PCR was performed to determine the changes in mRNA levels of Relm-α(A), Arg-1(B), CD206(C) and Ym-1(D) from lung tissues. The levels of mRNA are represented as the ratio to β-actin. n = 5–8 for each group. * indicates *p* < 0.05, ** indicates *p* < 0.01.

**Fig 6:**
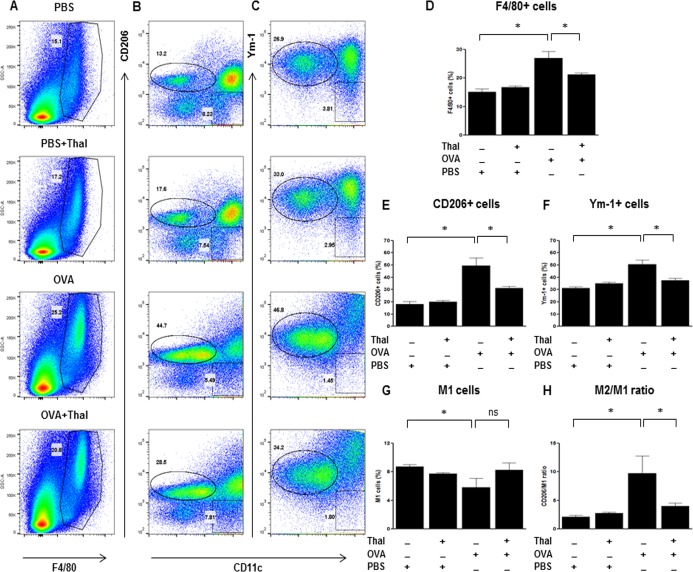
Effects of thalidomide on the populations of M2 macrophage markers in mouse lung tissues. A-C, Representative dot plot shows the expression of F4/80(A) in CD45+ cells from lung and CD206(B) and Ym-1(C) in F4/80+ macrophage cells. D-F, The frequencies of total F4/80+ macrophage cells(D) in CD45+ cells, CD206(E), and Ym-1(F) producing F4/80+ macrophage cells are shown. G-H, The levels of M1 (F4/80+ CD11c+CD206-) cells are represented as the frequencies(G) and the CD206 /M1 cells ratio(H) are represented. n = 5–8 for each group. * indicates *p* < 0.05

We explored the effects of thalidomide on change of M1 and M2 macrophage markers in lung homogenates. The level of Relm-α, Arg-1, CD206, and Ym-1 mRNA expressions from lung was significantly reduced when treated with thalidomide in OVA challenged mice (p < 0.05) (Fig 5A–5D). Flow cytometry performed on the lung homogenates revealed the following results. The frequency of F4/80 expressions in CD45+ cells from lung increased in OVA challenged mice but those populations were significantly reduced with thalidomide treatment (p < 0.05) (Fig 6A and 6D). Similarly, the frequency of CD206 and Ym-1 expressions in F4/80+ macrophage cells increased in OVA challenged mice but showed significant decrease with thalidomide treatment (p < 0.05) (Fig 6B–6C and 6E–6F). On the contrary, M1 macrophages by gating on F4/80+CD11c+CD206- cell [17,18] were reduced in OVA challenged mice compared control mice (p < 0.05) (Fig 6G). With thalidomide treatment, this population showed a recovering tendency but the difference was not significant compared to thalidomide untreated OVA challenged mice. As a result, the high M2/M1 ratio in OVA challenged mice significantly decreased with thalidomide treatment (Fig 6H).

**Fig 7:**
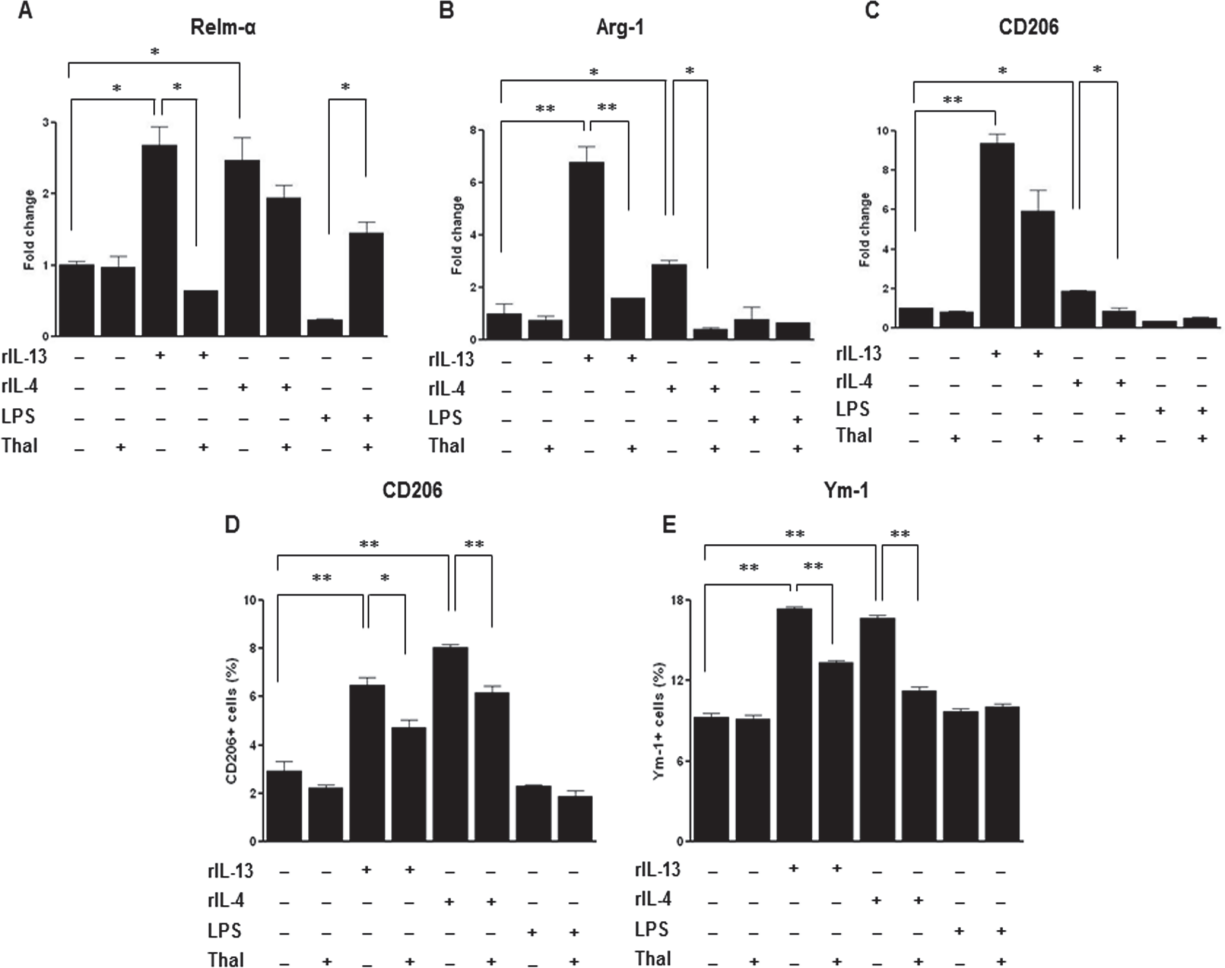
Effects of thalidomide on the expressions of M2 macrophage markers in mouse alveolar macrophages cell line. A-C, The mRNA levels of Relm-α(A), Arg-1(B), and CD206(C) were detected in Th2 cytokines stimulated mouse alveolar macrophages cell line. The levels of mRNA are represented as the ratio to β-actin. D-E, The frequencies of CD206(D) or Ym-1(E) were detected in Th2 cytokines stimulated mouse alveolar macrophages cell line using Flow cytometry. * indicates *p* < 0.05, ** indicates *p* < 0.01.

We also examined the *in vitro* effects of thalidomide on Th2 cytokines-skewed M2 macrophage markers using CRL-2456 macrophages. The mRNA expressions Relm-α, Arg-1, and CD206 showed significant decrease in IL-4 or IL-13 stimulated CRL-2456 macrophages with treatment of thalidomide (p < 0.05, p < 0.01) (Fig 7A–7C). Thalidomide treatment also resulted in a significant reduction in the frequencies of CD206 or Ym-1 expressing CRL-2456 macrophages induced by stimulation of IL-4 or IL-13 (Fig 7D and 7E).

### Discussion

Thalidomide was synthesized in 1954 by CIBA Pharmaceutical Company and widely prescribed as an antiemetic for morning sickness. However, it was withdrawn from most countries because of its high risk for teratogenicity. Recently, clinical investigations of thalidomide have been conducted in patients with diverse diseases, including multiple myeloma, rheumatoid arthritis, and human immunodeficiency virus infection [19,20] as the beneficial effects of thalidomide including anti-angiogenic, anti-viral, anti-inflammatory, and immunomodulatory activities were revealed [21]. Thalidomide shows anti-inflammatory activities, such as blocking TNF-α production by stimulated human peripheral blood mononuclear cells (PBMCs) [22] and by alveolar macrophages [23,24] and inhibiting IL-12 release [25].

Pharmacokinetic studies in healthy human male volunteers suggest a one-compartment, first order absorption and elimination model, with peak plasma levels reached within 4–5 hr and a half-life of 8.7 ± 4.1 hr [26]. In mice, peak plasma concentration of thalidomide after oral administration was attained after 1.0 hr when administered at 20 mg/kg and elimination half-life was 1.2±0.05 hr. In this study, thalidomide by oral administration (200 mg/kg) twice a day during challenge period could suppress eosinophil dominant airway inflammation in OVA-challenged mice.

Th2 cytokines such as IL-4, IL-5 and IL-13 are key mediators for the development of allergic airway inflammation and AHR. Recently, it has been shown that Th17 cells significantly enhance not only Th2-cell–mediated eosinophilic airway inflammation in airways but also Th2 cytokines-induced AHR [27]. TNF-α, which is also produced by Th17 cells [28], has been shown to induce eotaxin production from epithelial cells [29]. The present study demonstrates the attenuation of the number of inflammatory cells, mucin secretion, and cytokine levels of IL-4, IL-5 and IL-17 in BALF, the frequency of IL-4 and IL-5 producing CD4+ T cells in lung draining lymph nodes and spleen, and AHR to methacholine in OVA-challenged mice by thalidomide. Whereas all these Th2 changes were reduced with thalidomide treatment, serum IgG2 level and the frequency of IFN-γ from spleen cells increased in thalidomide treated mice compared to OVA group mice. Considering these findings, thalidomide seems to play a role in Th1/Th2 polarization in OVA asthma model. In addition, thalidomide also suppressed the expressions of TNF-α, IL-13, Eotaxin-1, and MUC-5AC in lung. Previous study showed that thalidomide had a limited suppressive effect on the expression of IL-5 and TNF-α and AHR, but not on IL-4 and IL-13 in OVA challenged mice [15]. Considering the relatively short half-life serum levels of the thalidomide in mice (≅1.2 hr in 20 mg/ kg dose) [16], the dose and frequency of thalidomide administration could significantly affect the effectiveness of the thalidomide in *in vivo* setting. Previously we and Asano, et al. administrated thalidomide once daily during each OVA challenge [14,15]. Since thalidomide was administered twice daily (before and after allergen challenge) in this study, we could have more profound and consistent *in vivo* effect of thalidomide.

The role of macrophages in the pathogenesis of asthma and allergic inflammation is still unclear. Recently, it is known that the Th2 cytokine profile in asthmatic lung contributes to the appearance of alternatively activated macrophages which were recruited into the lung following OVA sensitization and challenge [30]. These M2 subsets transferred to naive mice increase AHR, eosinophilic inflammation, and Th-2 cytokine secretion. Recently, it was reported that several mediators from M2 macrophages could contribute to promoting Th2 responses and suppressing Th1-driven inflammatory pathology [31,32]. A human study also demonstrated that M2 macrophages from human asthmatic patients directly contribute to the proliferation of CD4+ lymphocytes [33]. Significant up-regulation of M2 markers was reported in OVA- sensitized and-challenged mice, including Arg-1, MGL-2, Ym-1, Relm-α, and IL-10 [34]. Up-regulation of Arg-1 may be of particular physiologic importance. In patients with asthma, Arg-1 mRNA expression is increased in sub-mucosal inflammatory cells [35]. Arginase expression is increased in the lungs of allergen-sensitized and-challenged mice, and inhibition of its expression attenuates airway responsiveness to methacholine in OVA-sensitized and-challenged mice [36]. The role of Relm-α in Th2-driven pulmonary disease is known to contribute to the pathology of allergic airway disease, including airway remodeling and angiogenic responses [37,38]. Additionally, Ym-1 has been implicated in experimental and clinical asthma [39]. CD206, macrophage mannose receptor 1, is the first known marker of alternative macrophage activation, is related with fibrogenic condition and found to be up-regulated at the surface of alveolar macrophages from patients with idiopathic pulmonary fibrosis [40]. CD206 in human circulating fibrocytes was reported to have a role in regulation of allergen induced allergic response in asthma [41] but its role macrophage is not clearly investigated in asthma yet.

The differentiation of M2 macrophages is mediated by IL-4—and IL-13–dependent activation of STAT6, which leads to the expression of M2 markers such as Relm-α, and arginase in macrophages *in vitro* [42]. In this study, the administration of thalidomide to OVA-challenged mice attenuated the expression of M2 marker, such as Relm-α, Ym-1 and CD206, which are the major products of alternatively activated macrophages. Flow cytometry analysis for the evaluation of lung macrophage subsets reconfirmed reduction of M2 macrophage population but not M1 macrophage population by thalidomide. M1 macrophage population by gating on CD11c+CD206- cells and CD11c+Ym-1- cells showed rather a tendency to increase than to decrease. Based on these findings, thalidomide seems to inhibit polarization of M2 macrophages activated by Th2 stimulation but further study is needed to verify steps which thalidomide had an effect on. In support of *in vivo* finding, our *in vitro* studies also showed suppression of IL-4 or IL-13 induced mRNA expression of Relm-α, arginase-1, and CD206 on lung and CD206 or Ym-1 expressing macrophage population by thalidomide. These findings suggest the direct inhibitory effect of thalidomide on macrophage activation as well as Th2 responses could be a potential mechanism underlying anti-asthmatic effect of thalidomide shown in this study.

Last but not the least, the safety issue of thalidomide should be considered before application to human asthmatics. Thalidomide has had serious adverse effects when administered during pregnancy so its use cannot be justified for patients with asthma in general. However, it can be a possible candidate drug for some asthmatic patients who are refractory to traditional anti-asthmatic medication.

In conclusion, this study demonstrated that thalidomide was clearly effective in suppressing all features of allergic airway disease shown in OVA-challenged mice, including airway resistance to methacholine, inflammation, altered levels of allergen-specific IgG1/IgG2a, and the increased expression of proinflammatory cytokines and mediators associated with allergic Th2 inflammation. Thus, these studies highlight a potential use of thalidomide in the treatment of allergic diseases including asthma. This study further identified a novel inhibitory function of thalidomide on allergen- or Th2-cytokine stimulated alternative activation of macrophages as a potential mechanism of anti-asthmatic effect of thalidomide.

## References

[1] Cohn L, Tepper JS, Bottomly K. IL-4-independent induction of airway hyperresponsiveness by Th2, but not Th1, cells. J Immunol. 1998;161: 3813–3816. 9780144

[2] Vargaftig BB, Singer M. Leukotrienes, IL-13, and chemokines cooperate to induce BHR and mucus in allergic mouse lungs. Am J Physiol Lung Cell Mol Physiol. 2003;284: L260–269. 12388339 10.1152/ajplung.00226.2002

[3] Kay AB. The role of T lymphocytes in asthma. Chem Immunol Allergy. 2006;91: 59–75. 16354949 10.1159/000090230

[4] Corrigan CJ, Hartnell A, Kay AB. T lymphocyte activation in acute severe asthma. Lancet. 1988;1: 1129–1132. 2896958 10.1016/s0140-6736(88)91951-4

[5] Walker C, Kaegi MK, Braun P, Blaser K. Activated T cells and eosinophilia in bronchoalveolar lavages from subjects with asthma correlated with disease severity. J Allergy Clin Immunol. 1991;88: 935–942. 1744364 10.1016/0091-6749(91)90251-i

[6] Zhu Z, Zheng T, Homer RJ, Kim YK, Chen NY, Cohn L, et al Acidic mammalian chitinase in asthmatic Th2 inflammation and IL-13 pathway activation. Science. 2004;304: 1678–1682. 15192232 10.1126/science.1095336

[7] Nair MG, Cochrane DW, Allen JE. Macrophages in chronic type 2 inflammation have a novel phenotype characterized by the abundant expression of Ym1 and Fizz1 that can be partly replicated in vitro. Immunol Lett. 2003;85: 173–180. 12527225 10.1016/s0165-2478(02)00225-0

[8] Nair MG, Gallagher IJ, Taylor MD, Loke P, Coulson PS, Wilson RA, et al Chitinase and Fizz family members are a generalized feature of nematode infection with selective upregulation of Ym1 and Fizz1 by antigen-presenting cells. Infect Immun. 2005;73: 385–394. 15618176 10.1128/IAI.73.1.385-394.2005 PMC538942

[9] Vercelli D. Arginase: marker, effector, or candidate gene for asthma? J Clin Invest. 2003;111: 1815–1817. 12813015 10.1172/JCI18908 PMC161429

[10] Homer RJ, Zhu Z, Cohn L, Lee CG, White WI, Chen S, et al Differential expression of chitinases identify subsets of murine airway epithelial cells in allergic inflammation. Am J Physiol Lung Cell Mol Physiol. 2006;291: L502–511. 16556727 10.1152/ajplung.00364.2005

[11] Martinez FO, Helming L, Milde R, Varin A, Melgert BN, Draijer C, et al Genetic programs expressed in resting and IL-4 alternatively activated mouse and human macrophages: similarities and differences. Blood. 2013;121: e57–69. 10.1182/blood-2012-06-436212 23293084

[12] Kim EY, Battaile JT, Patel AC, You Y, Agapov E, Grayson MH, et al Persistent activation of an innate immune response translates respiratory viral infection into chronic lung disease. Nat Med. 2008;14: 633–640. 10.1038/nm1770 18488036 PMC2575848

[13] Boorsma CE, Draijer C, Melgert BN. Macrophage heterogeneity in respiratory diseases. Mediators Inflamm. 2013;2013: 769214 10.1155/2013/769214 23533311 PMC3600198

[14] Kwon HS, Lee HS, Kang HR, Min KU, Kim YY, Cho SH, et al. Effect of thalidomide on airway inflammation and airway hyperresponsiveness in mouse models of asthma. D.Ph. Thesis, The University of Seoul National. (Available: http://library.snu.ac.kr/search/DetailView.ax?sid=12&cid=3101342.

[15] Asano T, Kume H, Taki F, Ito S, Hasegawa Y. Thalidomide attenuates airway hyperresponsiveness and eosinophilic inflammation in a murine model of allergic asthma. Biol Pharm Bull. 2010;33: 1028–1032. 20522972 10.1248/bpb.33.1028

[16] Chung F, Lu J, Palmer BD, Kestell P, Browett P, Baguley BC, et al Thalidomide pharmacokinetics and metabolite formation in mice, rabbits, and multiple myeloma patients. Clin Cancer Res. 2004;10: 5949–5956. 15355928 10.1158/1078-0432.CCR-04-0421

[17] Han MS, Jung DY, Morel C, Lakhani SA, Kim JK, Flavell RA, et al JNK expression by macrophages promotes obesity-induced insulin resistance and inflammation. Science. 2013;339: 218–222. 10.1126/science.1227568 23223452 PMC3835653

[18] Kim HY, Lee HJ, Chang YJ, Pichavant M, Shore SA, Fitzgerald KA, et al Interleukin-17-producing innate lymphoid cells and the NLRP3 inflammasome facilitate obesity-associated airway hyperreactivity. Nat Med. 2014;20: 54–61. 10.1038/nm.3423 24336249 PMC3912313

[19] Calabrese L, Fleischer AB. Thalidomide: current and potential clinical applications. Am J Med. 2000;108: 487–495. 10781782 10.1016/s0002-9343(99)00408-8

[20] Kamikawa R, Ikawa K, Morikawa N, Asaoku H, Iwato K, Sasaki A. The pharmacokinetics of low-dose thalidomide in Japanese patients with refractory multiple myeloma. Biol Pharm Bull. 2006;29: 2331–2334. 17077542 10.1248/bpb.29.2331

[21] Hashimoto Y. Thalidomide as a multi-template for development of biologically active compounds. Arch Pharm (Weinheim). 2008;341: 536–547. 10.1002/ardp.200700217 18389516

[22] Sampaio EP, Sarno EN, Galilly R, Cohn ZA, Kaplan G. Thalidomide selectively inhibits tumor necrosis factor alpha production by stimulated human monocytes. J Exp Med. 1991;173: 699–703. 1997652 10.1084/jem.173.3.699 PMC2118820

[23] Tavares JL, Wangoo A, Dilworth P, Marshall B, Kotecha S, Shaw RJ. Thalidomide reduces tumour necrosis factor-alpha production by human alveolar macrophages. Respir Med. 1997;91: 31–39. 9068814 10.1016/s0954-6111(97)90134-7

[24] Moreira AL, Sampaio EP, Zmuidzinas A, Frindt P, Smith KA, Kaplan G. Thalidomide exerts its inhibitory action on tumor necrosis factor alpha by enhancing mRNA degradation. J Exp Med. 1993;177: 1675–1680. 8496685 10.1084/jem.177.6.1675 PMC2191046

[25] Moller DR, Wysocka M, Greenlee BM, Ma X, Wahl L, Flockhart DA, et al Inhibition of IL-12 production by thalidomide. J Immunol. 1997;159: 5157–5161. 9366446

[26] Chen TL, Vogelsang GB, Petty BG, Brundrett RB, Noe DA, Santos GW, et al Plasma pharmacokinetics and urinary excretion of thalidomide after oral dosing in healthy male volunteers. Drug Metab Dispos. 1989;17: 402–405. 2571480

[27] Wakashin H, Hirose K, Maezawa Y, Kagami S, Suto A, Watanabe N, et al IL-23 and Th17 cells enhance Th2-cell-mediated eosinophilic airway inflammation in mice. Am J Respir Crit Care Med. 2008;178: 1023–1032. 10.1164/rccm.200801-086OC 18787221

[28] Langrish CL, Chen Y, Blumenschein WM, Mattson J, Basham B, Sedgwick JD, et al IL-23 drives a pathogenic T cell population that induces autoimmune inflammation. J Exp Med. 2005;201: 233–240. 15657292 10.1084/jem.20041257 PMC2212798

[29] Lilly CM, Nakamura H, Kesselman H, Nagler-Anderson C, Asano K, Garcia-Zepeda EA, et al Expression of eotaxin by human lung epithelial cells: induction by cytokines and inhibition by glucocorticoids. J Clin Invest. 1997;99: 1767–1773. 9120022 10.1172/JCI119341 PMC507998

[30] Moon KA, Kim SY, Kim TB, Yun ES, Park CS, Cho YS, et al Allergen-induced CD11b+ CD11c(int) CCR3+ macrophages in the lung promote eosinophilic airway inflammation in a mouse asthma model. Int Immunol. 2007;19: 1371–1381. 17977814 10.1093/intimm/dxm108

[31] Donnelly S, Stack CM, O'Neill SM, Sayed AA, Williams DL, Dalton JP. Helminth 2-Cys peroxiredoxin drives Th2 responses through a mechanism involving alternatively activated macrophages. FASEB J. 2008;22: 4022–4032. 10.1096/fj.08-106278 18708590 PMC3980656

[32] La Flamme AC, Kharkrang M, Stone S, Mirmoeini S, Chuluundorj D, Kyle R. Type II-activated murine macrophages produce IL-4. PLoS One. 2012;7: e46989 10.1371/journal.pone.0046989 23071691 PMC3465319

[33] Winkler C, Witte L, Moraw N, Faulenbach C, Muller M, Holz O, et al Impact of endobronchial allergen provocation on macrophage phenotype in asthmatics. BMC Immunol. 2014;15: 12 10.1186/1471-2172-15-12 24612750 PMC4007705

[34] Nagarkar DR, Bowman ER, Schneider D, Wang Q, Shim J, Zhao Y, et al Rhinovirus infection of allergen-sensitized and-challenged mice induces eotaxin release from functionally polarized macrophages. J Immunol. 2010;185: 2525–2535. 10.4049/jimmunol.1000286 20644177 PMC3208235

[35] Zimmermann N, King NE, Laporte J, Yang M, Mishra A, Pope SM, et al Dissection of experimental asthma with DNA microarray analysis identifies arginase in asthma pathogenesis. J Clin Invest. 2003;111: 1863–1874. 12813022 10.1172/JCI17912 PMC161427

[36] North ML, Khanna N, Marsden PA, Grasemann H, Scott JA. Functionally important role for arginase 1 in the airway hyperresponsiveness of asthma. Am J Physiol Lung Cell Mol Physiol. 2009;296: L911–920. 10.1152/ajplung.00025.2009 19286931

[37] Dong L, Wang SJ, Camoretti-Mercado B, Li HJ, Chen M, Bi WX. FIZZ1 plays a crucial role in early stage airway remodeling of OVA-induced asthma. J Asthma. 2008;45: 648–653. 10.1080/02770900802126941 18951255

[38] Sun Y, Wang J, Li H, Han X. Found in inflammatory zone 1 induces angiogenesis in murine models of asthma. Lung. 2008;186: 375–380. 10.1007/s00408-008-9099-1 18758859

[39] Chupp GL, Lee CG, Jarjour N, Shim YM, Holm CT, He S, et al A chitinase-like protein in the lung and circulation of patients with severe asthma. N Engl J Med. 2007;357: 2016–2027. 18003958 10.1056/NEJMoa073600

[40] Pechkovsky DV, Prasse A, Kollert F, Engel KM, Dentler J, Luttmann W, et al Alternatively activated alveolar macrophages in pulmonary fibrosis-mediator production and intracellular signal transduction. Clin Immunol. 2010;137: 89–101. 10.1016/j.clim.2010.06.017 20674506

[41] Tsai YM, Hsu SC, Zhang J, Zhou YF, Plunkett B, Huang SK, et al Functional interaction of cockroach allergens and mannose receptor (CD206) in human circulating fibrocytes. PLoS One. 2013;8: e64105 10.1371/journal.pone.0064105 23734186 PMC3667076

[42] Mikita T, Campbell D, Wu P, Williamson K, Schindler U. Requirements for interleukin-4-induced gene expression and functional characterization of Stat6. Mol Cell Biol. 1996;16: 5811–5820. 8816495 10.1128/mcb.16.10.5811 PMC231582

